# Double Perovskite Ba_2_LaTaO_6_ for Ultrafast Fiber Lasers in Anomalous and Normal Net Dispersion Regime

**DOI:** 10.3390/nano12122112

**Published:** 2022-06-20

**Authors:** Yiqing Shu, Mingqi An, Penglai Guo, Xun Yuan, Leiming Wu, Zhitao Lin, Weicheng Chen, Xiaohui Li, Jianqing Li

**Affiliations:** 1 School of Computer Science and Engineering, Macau University of Science and Technology, Avenida Wai Long, Taipa, Macao, China; 1909853yii30001@student.must.edu.mo (Y.S.); penglai_guo@163.com (P.G.); metabutterflyjun@gmail.com (X.Y.); 1809853gii30001@student.must.edu.mo (Z.L.); 2 Guangdong-HongKong-Macao Joint Laboratory for Intelligent Micro-Nano Optoelectronic Technology, Foshan University, Foshan 528225, China; 3 School of Physics and Information Technology, Shaanxi Normal University, Xi’an 710119, China; 17543068643@163.com; 4 Institute of Advanced Photonics Technology, School of Information Engineering, Guangdong University of Technology, Guangzhou 510006, China; leiming_wu@gdut.edu.cn

**Keywords:** double perovskite oxide, Ba_2_LaTaO_6_, nonlinear optical response, anomalous and normal dispersion region, ultrafast fiber laser

## Abstract

Double perovskites (DPs) have been attracting attention in an assortment of optoelectronic applications, for they hold advantages such as high quantum efficiency, long carrier migration distance and strong linear and nonlinear absorptions. As specific kinds of perovskites (PVKs), DPs are gifted with orthorhombic crystal structures which provide rich conversion combinations and broaden the space for research and application. However, few works have been reported about DPs in ultrafast photonics applications. In this article, a DP with chemical formula of Ba_2_LaTaO_6_ (BLT) was successfully synthesized by high-temperature solid phase method. The microstructures and morphologies were observed, and the linear and nonlinear absorption were characterized. By first using BLT as a novel saturable absorber in both normal and anomalous dispersion region fiber lasers, dual-wavelength soliton and dissipative soliton were successfully operated at C-band. This study affirms BLT’s nonlinear optical properties, lays the foundation for optical research on BLT, and meanwhile provides a meaningful reference for future development of pulsed lasers based on DPs.

## 1. Introduction

Perovskites (PVKs) are a family of ceramic-like oxides with a general chemical formula of ABX_3,_ where the A-site is occupied by a cation with a large ionic radius (Ba, Sr, Ca and so on); the B-site represents another cation of a transitional metal or a rare earth element, and X represents an anion, usually oxygen or a halogen [1,2,3,4,5]. Double perovskites (DPs) are a specific group in the family, with a doubled chemical structure of A_2_B’B”O_6_ and can be obtained by adjusting the composition of foresaid PVKs [6,7]. They possess stable skeletal structures and the characteristics can be engineered by changing the species and ratio of elemental components [8]. Due to their tunable electrical and optical properties of high internal quantum efficiency, long charge collection distance and strong linear and nonlinear absorptions, DPs have received wide attention for promising applications in solar cells and optoelectronic devices such as light emitting diodes, light modulators, photodetectors, phototransistors and lasers [9,10,11,12,13]. As one of the potentially attractive compounds of the DP family, a kind of barium lanthanide tantalates with a chemical formula of Ba_2_LaTaO_6_ (BLT) and an orthorhombic crystal structure was first reported by Galasso et al. in 1966 [14,15]. As the research deepened, some conspicuous properties of BLT in electronics, magnetism and optics were gradually revealed. In 2001, Doi et al. reported BLT was Van Vleck paramagnetic down to 5 K. This led to application potentials in the direction of electron paramagnetic resonance imaging [16]. In 2006, Korchagina et al. reported the characterization of BLT and confirmed the relationship of its low-frequency and microwave dielectric properties with the ionic radii of constituent lanthanide cations [17]. In 2014, Kumari et al. successfully obtained the bandgap of BLT as 2.03 eV and the dielectric relaxation described an unchanged mechanism at different temperatures [14]. These findings have suggested the applications of BLT in nonlinear optical (NLO) devices.

Nonlinear optics is the study on the nonlinear response of matter under intense coherent light and the applications therefrom [18,19,20]. The research of NLO materials is of great significance to the development of laser technology, spectroscopy, and the analysis of material structures [21,22,23,24]. PVKs are a group of often seen NLO materials with tunable bandgap, remarkable exciton properties at room temperature, high quantum yield and are of low cost [9,12,18]. Moreover, their chemical and structural diversity makes it possible to engineer their nonlinear absorption and nonlinear refraction features [18,25]. Hence, PVKs are of advantageous potential in NLO applications and are considered as promising saturable absorber (SA) candidates [26,27]. In 2016, Zhang et.al. demonstrated a pulsed laser with the Organic–Inorganic Halide Perovskites (CH_3_NH_3_PbI_3–x_Cl_x_) as an SA, indicating the potentials of PVKs to be employed in nonlinear optoelectronic devices [28]. In 2018, Hong et al. reported an all-fiber laser operating in the C- and L-bands using (C_6_H_5_C_2_H_4_NH_3_)_2_PbI_4_ PVK crystallites as an SA, showing the potentials of hybrid organic–inorganic PVKs in nonlinear photonics in a wide spectral range [29]. In 2021, Li et al. presented the saturable absorption properties of CsPbBr_3_ PVK quantum dots in a passively *Q*-switched visible solid-state laser [30]. Much attention has been drawn as the studies on PVKs continuously increase and more than 3000 manuscripts have been published since 2017 [8,25,31,32,33,34,35,36]. BLT, a seasoned member in the family, however, is seemingly forgotten and research remains on a primary stage in regard to optics, due to tantalum being scarce and the synthesis methods yet to be improved.

In this study, BLT was successfully synthesized by high-temperature solid phase method [14]. The typical microstructure of BLT was characterized, and the nonlinear optical response was investigated using two-arm detection method [37,38]. The saturation intensity and modulation depth of BLT were 2.96 MW/cm^2^ and 18.6%, respectively. To further evaluate its performance and potentials in optical devices, for the first time, BLT was utilized as an SA integrated into two C-band fiber lasers operating in normal and anomalous dispersion region sequentially. The generation of a conventional soliton with pulse duration time of 1.3 ps and a dissipative soliton with pulse duration time of 55.8 ps was successfully achieved. Additionally, after slightly rotating the polarization controller (PC) in the anomalous dispersion fiber laser, a dual-wavelength soliton was obtained [39]. These results suggest that BLT can be used as a new type of NLO material for various nonlinear optics and photonic applications, and meanwhile lay the foundation for optical research on BLT and provide a meaningful reference for the development of pulsed lasers based on DPs.

## 2. Synthesis and Characterization

**Figure 1:**
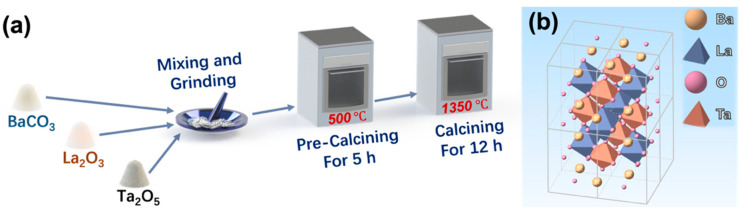
(**a**) Synthesis process of BLT. (**b**) Crystal structure schematic of BLT.

BLT was synthesized using a more efficient method, an optimized version of previously reported high-temperature synthesis method that greatly reduces the synthesis time [14,16,17]. The detailed preparation process is given in Figure 1a, and the chemical reaction equation is as follows:

4 BaCO_3_ + La_2_O_3_ + Ta_2_O_5_ → 2 Ba_2_LaTaO_6_ + 4 CO_2_

As shown in Figure 1a, BaCO_3_ (99.9%, 0.002 mol, Sinopharm Chemical Reagent Co., Ltd, Shanghai, China), La_2_O_6_ (99.99%, 0.0005 mol, Sinopharm Chemical Reagent Co., Ltd.), and Ta_2_O_5_ (99.99%, 0.0005 mol, Sinopharm Chemical Reagent Co. Ltd) were mixed and ground with a pestle in an agate mortar for 20 min in order to increase the contact area. The mixture was pre-calcined in a high-temperature muffle furnace at 500 °C for 5 h for purification, then calcined at 1350 °C for 12 h again. The calcined product was cooled down to room temperature and ground to powder, thus the target produce BLT was obtained. Its crystal structure schematic is shown in Figure 1b, in which B’-site (La) and B”-site (Ta) atoms forms chemical bonds of B’O_6_ and B”O_6_ with nearest oxygen atoms to constitute regular octahedral structures [14].

**Figure 2:**
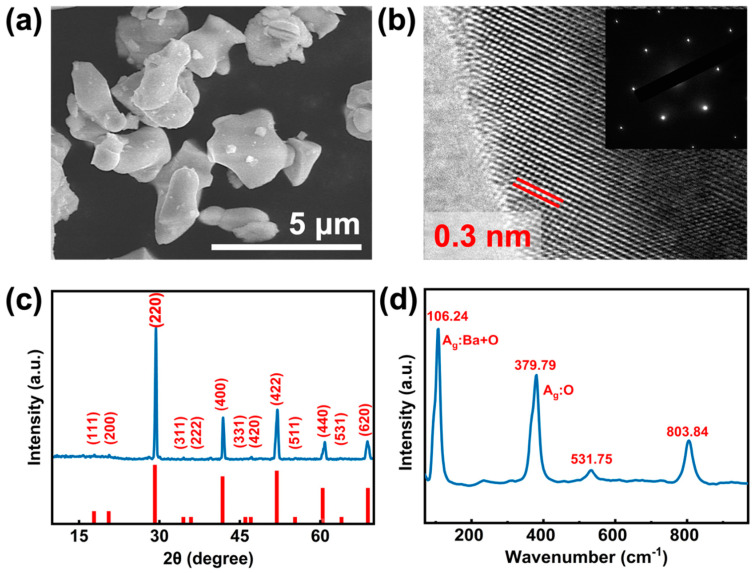
Morphology and characterization of the prepared BLT. (**a**) SEM image. (**b**) HR-TEM image. Inset: SEAD image. (**c**) Measured and standard XRD pattern. (**d**) Raman Spectrum.

The microscopic morphological characteristics of the prepared BLT were determined by scanning electron microscope (SEM, Nova Nano-SEM450, FEI Company, Brno, Czech Republic); clearly, the BLT particles are well dispersed, as Figure 2a shows, in the span of 5 μm. High-resolution transmission electron microscopy (HR-TEM, Tecnai G2 F20, FEI Company) and selected area electron diffraction (SAED, Hitachi SU-8010, Tokyo, Japan) images reveal the typical micro-crystal structures of BLT [14], as shown in Figure 2b. The measured lattice distance of BLT is about 0.3 nm, close to a theoretical value of 3.06 Å in (220) plane [17]. The typical monocrystalline structure is displayed in the inset of Figure 2b, conforming with the earlier research [14,17]. To further identify the quality of BLT, X-ray diffraction (XRD, Rigaku-Dmax 3C, Tokyo, Japan) data were obtained, as exhibited in Figure 2c. The peaks located at 14.58° (220), 20.89° (400), 25.96° (422), 30.23° (440), 34.44° (620), correspond perfectly with the standard pattern, inferring that the sample was desired BLT with high purity. Additionally, the identity of the prepared sample was verified with Raman spectroscopy (LabRam HR Evolution Raman, Horiba, Kyoto, Japan) at room temperature. As presented in Figure 2d, 2 obvious characteristic Raman peaks are located at 106.24 cm^−1^ and 379.79 cm^−1^, corresponding to the active mode of Ag (vibrating atoms: Ba + O) and Ag (vibrating atoms: O), respectively. These results consist with previous findings and suggest that BLT was successfully prepared with high quality.

**Figure 3:**
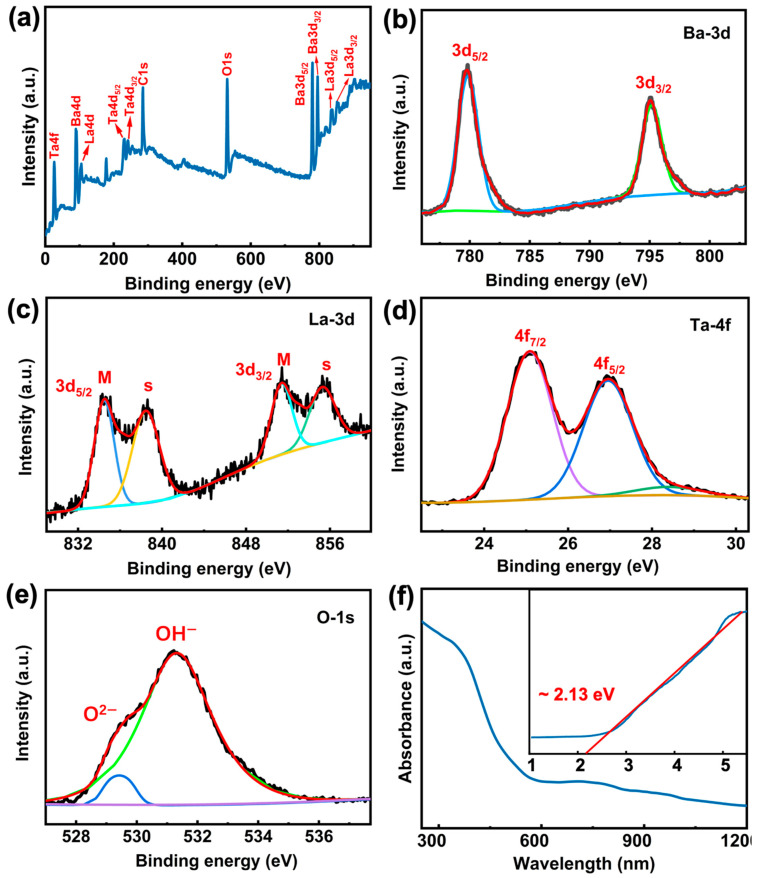
XPS spectra and UV–VIS–NIR of prepared BLT. (**a**) Survey scan of XPS spectrum. (**b**–**e**) HR-XPS spectrum. (**f**) UV–vis–NIR absorption spectrum. Inset: Tauc analysis of UV-VIS-NIR absorption.

To discern the element components and energy states in the prepared BLT, high-resolution X-ray photoelectron spectroscopy (XPS, AXIS-ULTRA DLD, Kratos, Manchester, UK) was implemented. As shown in Figure 3a, Ba-3d, La-3d, La-4d, Ta-4d, Ta-4f and O-1s are identified and indexed in a wide scan range of XPS. The high-resolution core-level spectra of above elements are described in Figure 3b–e to confirm the oxidation states of the constituent ions. The Ba-3d spectrum, in Figure 3b, splits into 2 components of 3d_5/2_ and 3d_3/2_ due to the spin-orbit interaction. The La-3d spectrum, in Figure 3c, has 2 doublets in the two peaks of 3d_5/2_ and 3d_3/2_ caused by the multiple splitting [14]. The Ta-4f state, as shown in Figure 3d, is represented by 2 peaks of 4f_7/2_ and 4f_5/2_. Additionally, the 2 peaks in the O-1s spectrum are bounded up with the presence of 2 distinct oxygen sites in the BLT crystal structure at Figure 3e [14]. The linear optical absorption from visible light to near infrared of BLT was obtained via an ultraviolet-visible-near infrared (UV-VIS-IR, Carry-5000 UV-vis, Agilent Technologies, Santa Clara, CA, USA) spectrometer as depicted in Figure 3f. As the BLT dispersions were caused by the ground product with different particle sizes and shapes, the absorption spectrum is the result of the superposition of said particles’ absorption spectra, which smoothly changes with increasing wavelength from 300 nm to 1200 nm. The optical bandgap is calculated to be ~2.13 eV with the Tauc method as shown in inset of Figure 3f. This is very close to the previous published work of 2.03 eV [14]. These characterization data demonstrate the successful preparation of high-quality BLT and the broadband linear optical response of BLT; furthermore, they provide an expectation for its NLO applications in the field of optics as a photonics device.

## 3. Nonlinear Optical Response

**Figure 4:**
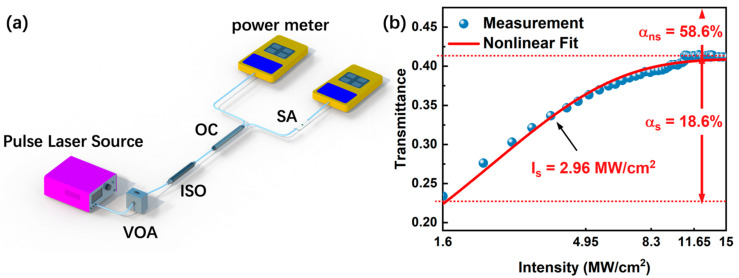
(**a**) The schematic of saturable absorption measurement system. (**b**) Nonlinear optical response of BLT SA.

The mechanism of saturable absorption, an important NLO response, is believed to be the Pauli blocking principle [22,25]. In semiconductors, electrons in the valence band can be stimulated by light beams to migrate to the conduction band. When the conduction band is completely filled with the excited electrons, the material will stop absorbing photons and the incoming photons will pass through the material [40]. Such materials are called SAs [41,42]. SAs are widely applied in pulsed lasers as mode lockers and their NLO response properties are concerned tightly with the lasers’ performance [43,44]. The optical induced deposition method was used to fabricate a BLT SA by depositing BLT onto the surface of the waist area in a tapered fiber and the NLO response of the BLT SA was investigated by balanced twin detection. The schematic of the saturable absorption measurement system is exhibited in Figure 4a. The pump laser injected into a variable optical attenuator (VOA) to adjust its output power without changing the shape of the pulse. A 50/50 optical coupler (OC) divided the laser into two individual beams, one beam was detected directly as a reference light by a power meter, and the other one was detected as a signal light by another power meter after travelling through the SA for NLO response occurrence. The experimental data was fitted using the SA model function [45]:

(1) $$T\left(I\right)=1-\frac{\alpha_{s}}{1+\frac{I}{I_{s}}}-\alpha_{ns}$$

where *T(I)* is transmittance, *α_s_*, *α_ns_*, *I* and *I_s_* are modulation depth, non-saturable loss, laser power intensity and saturation intensity, respectively. As presented in Figure 4b, the dependence of absolute transmission of BLT SA on its corresponding incident peak intensity are clearly revealed. The *α_s_* and *I_s_* of the SA were fitted to be 18.6% and 2.96 MW/cm^2^, respectively. These conclusions illustrate untapped potentials of BLT as an NLO material in the application as photonics devices.

## 4. Ultrafast Photonics Application

**Figure 5:**
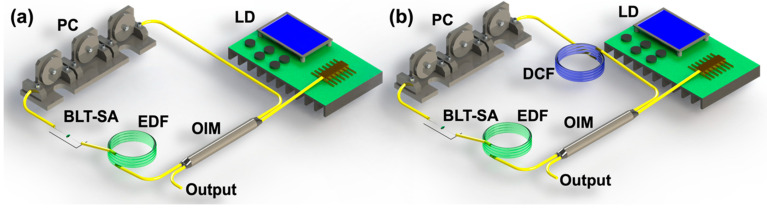
Schematic diagram of all-fiber laser cavities with net (**a**) anomalous dispersion and (**b**) normal dispersion.

Thanks to the broadband linear optical response and NLO characteristics of BLT, the BLT SA was considered to be integrated into all-fiber ring laser cavities of anomalous and normal net dispersion, respectively. Detailed configurations of the laser cavities are illustrated in Figure 5. Both lasers possess similar structures including a pump, an optical integrated module (OIM), a 0.3-m-long erbium-doped gain fiber (EDF), a polarization controller (PC) and a BLT-SA. The net dispersion of the lasers is discrepant and controlled by changing the length of dispersion control fiber (DCF). The net cavity dispersion value was located in an anomalous dispersion region without integrating the DCF and converted to normal dispersion region when sufficient DCF was inserted into the cavity. By adjusting the pump power and polarization states via rotating the angle of the PC, various kinds of ultrashort pulses were steadily generated and their performances were systematically recorded.

### 4.1. Anomalous Dispersion Ultrashort Pulse in C-Band

**Figure 6:**
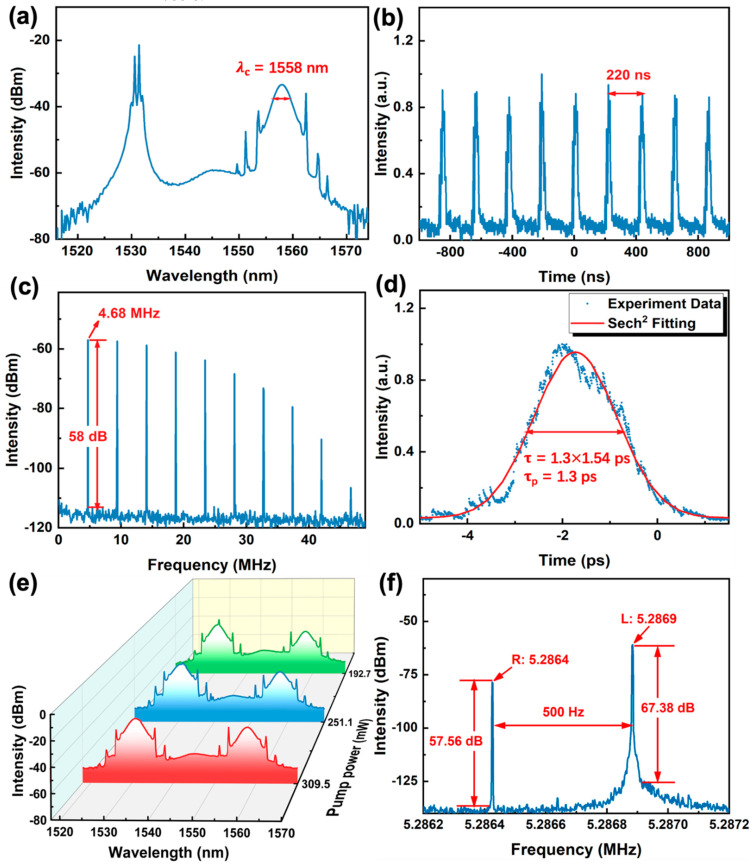
Anomalous dispersion ultrashort pulse output characteristics based on BLT SA. (**a**) Optical spectrum of conventional soliton. (**b**) The output pulse sequence of conventional soliton. (**c**) Broadband RF spectrum of conventional soliton. (**d**) Auto-correlation trace of conventional soliton. (**e**) Optical spectrum of dual-wavelength soliton. (**f**) Fine structure of RF spectrum of dual-wavelength soliton.

A typical conventional soliton, along with a continuous wave (CW) laser located at 1530 nm, was generated with central wavelength of 1558 nm and 3 dB bandwidth of 2.61 nm when the pump power was 175 mW, as displayed in Figure 6a. The pulse train with the pulse interval of 220 ns in the span of 2000 ns is exhibited in Figure 6b and presents a typical bright-soliton emission state. To further demonstrate the stability of the laser, the broadband RF spectrum was measured. As shown in Figure 6c, the signal-to-noise ratio (SNR) is about 58 dB and the repetition rate is located at the fundamental frequency of 4.68 MHz, corresponding to the cavity length of ~44 m, illustrating a good stability of this mode-locked pulse. The autocorrelation trace of the soliton was measured by a real-time oscilloscope and shown in Figure 6d. The pulse duration of 1.3 ps was obtained by fitting the data with squared hyperbolic secant (Sech^2^) profile [34]. The corresponding time-bandwidth product (*TBP*) of the soliton can be obtained by the following equation [40]:

(2) $$TBP=\tau_{pulse}\times c\cdot \frac{\Delta \lambda }{\lambda_{c}^{2}}$$

where *τ_pulse_*, *c*, Δ*λ*, and *λ_c_* represent the pulse duration, light speed, 3 dB bandwidth, and center wavelength of the optical spectrum. The *TBP* was calculated to be ~0.43 (>0.315), indicating a slight chirp in the laser cavity and hinting some optimization potentials to further compress the pulse duration.

Moreover, by keeping the rotation angle of the PC still and continuously increasing the pump power, dual-wavelength mode-locked pulses were observed. When the pump power reached 309.5 mW, the CW laser could no longer be observed, and the short wavelength is located at 1530.6 nm meanwhile the long wavelength is located at 1555.7 nm in the dual-wavelength spectrum, as exhibited in Figure 6e. The RF spectrum is presented in Figure 6f; the relationship between RF separation interval (Δf) and the different operation wavelength can be described by this formula [46]:

(3) $$\Delta f=\frac{c^{2}D\Delta \lambda }{n^{2}\left(L+LD\Delta \lambda \frac{c}{n}\right)}$$

where D and L are the cavity dispersion and cavity length, *c* and *n* are the velocity of light and the fiber refractive index, respectively. The RF peaks interval was calculated to be 500 Hz and consistent with the experimental data denoted in Figure 6f. These results verify that the BLT SA is a competitive candidate for realizing multiwavelength pulsed lasers.

### 4.2. Normal Dispersion Ultrashort Pulse in C-Band

**Figure 7:**
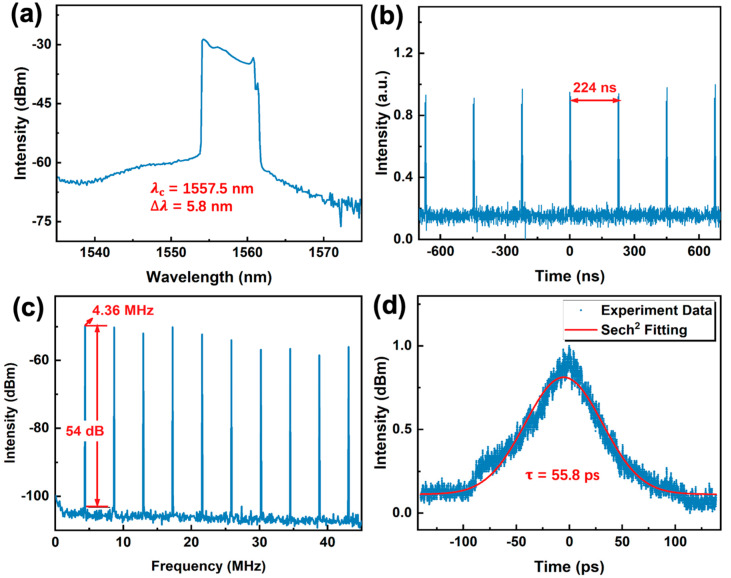
Normal dispersion ultrashort pulse output characteristics based on BLT SA. (**a**) Optical spectrum of dissipative soliton. (**b**) The output pulse sequence of dissipative soliton. (**c**) Broadband RF spectrum of dissipative soliton. (**d**) Auto-correlation trace of dissipative soliton.

Distinct from conventional solitons generated in anomalous dispersion regime fiber lasers, which can be described by the nonlinear Schrödinger equation (NLSE), solitons generated in normal dispersion regime can be analyzed using the Ginzburg–Landau equation (GLE) and are known as dissipative solitons (DSs) [47]. On account of their characteristics including larger pulse energy, wider pulse duration and greater chirp compared to NLSE soliton, DSs have been regarded as potential candidates for generating large energy ultra-short pulses [48]. To investigate the ultrashort pulse emission in normal dispersion regime based on the BLT SA, a segment of DCF of 7.5 m length was embedded into the laser cavity as presented in Figure 5b. By changing the pump power and the polarization state, a typical DS pulse, with a flat-top square-shaped spectrum as shown in Figure 7a, was generated. Its center wavelength is located at 1557.5 nm with the 3 dB spectral bandwidth of 5.8 nm. The real time trace of oscilloscope with a pulse interval of ~224 ns is depicted in Figure 7b. In the frequency domain, the RF spectrum shows the repetition rate of about 4.36 MHz and the SNR of 54 dB in Figure 7c. In the temporal domain, the autocorrelation trace was fitted with a sech^2^ profile, and the pulse duration was obtained to be 55.8 ps as shown in Figure 7d. To determine whether the mode-locked pulse generation was caused by the NLO response of BLT, the BLT SA was removed. No pulse generation was observed in the same condition. This clearly confirmed that the BLT SA was the key factor for ultrafast pulse generation, and further promotes the research on normal dispersion ultrashort pulse generation based on PVK materials.

## 5. Conclusions

In conclusion, a DP with the chemical formula of Ba_2_LaTaO_6_ were successfully synthesized using high-temperature solid-state method. Its micro morphology characterization was exhibited using SEM, HRTEM and SEAD, its high quality was confirmed using XRD, XPS and Raman spectroscopy, and strong broadband optical absorption of prepared BLT were measured. To further investigate the nonlinear absorption characteristics of BLT, an SA based on BLT was fabricated. Its saturation intensity of 2.96 MW/cm^2^ and modulation depth of 18.6% were investigated by a 2-arm detection method. Considering the potential of BLT as an NLO material, the BLT SA was integrated into normal and anomalous dispersion region fiber lasers, in succession, to investigate its ultrafast photonics applications. A stable conventional soliton and a dissipative soliton with pulse duration time of 1.3 ps and 55.8 ps were obtained at C-band, respectively. A dual-wavelength soliton was achieved in the anomalous dispersion regime fiber laser by increasing the pump power to 309.5 mW. These results affirm the excellent NLO properties and rich soliton emission behaviors of BLT, laying the foundation for future optical research and providing a meaningful reference for developing DP-based optical devices.

## Data Availability

Not applicable.
